# Effective impairment of myeloma cells and their progenitors by hyperthermia

**DOI:** 10.18632/oncotarget.23121

**Published:** 2017-12-07

**Authors:** Hirokazu Miki, Shingen Nakamura, Asuka Oda, Hirofumi Tenshin, Jumpei Teramachi, Masahiro Hiasa, Ariunzaya Bat-Erdene, Yusaku Maeda, Masahiro Oura, Mamiko Takahashi, Masami Iwasa, Takeshi Harada, Shiro Fujii, Kiyoe Kurahashi, Sumiko Yoshida, Kumiko Kagawa, Itsuro Endo, Kenichi Aihara, Mariko Ikuo, Kohji Itoh, Koichiro Hayashi, Michihiro Nakamura, Masahiro Abe

**Affiliations:** ^1^ Division of Transfusion Medicine and Cell Therapy, Tokushima University Hospital, Tokushima, Japan; ^2^ Department of Hematology, Endocrinology and Metabolism, Institute of Biomedical Sciences, Tokushima University Graduate School, Tokushima, Japan; ^3^ Department of Orthodontics and Dentofacial Orthopedics, Institute of Biomedical Sciences, Tokushima University Graduate School, Tokushima, Japan; ^4^ Department of Histology and Oral Histology, Institute of Biomedical Sciences, Tokushima University Graduate School, Tokushima, Japan; ^5^ Department of Biomaterials and Bioengineering, Institute of Biomedical Sciences, Tokushima University Graduate School, Tokushima, Japan; ^6^ Department of Medicinal Biotechnology, Institute for Medicinal Research, Graduate School of Pharmaceutical Science, Tokushima University, Tokushima, Japan; ^7^ Division of Materials Research, Institute of Materials and Systems for Sustainability, Nagoya University, Aichi, Japan; ^8^ Department of Organ Anatomy, Yamaguchi University Graduate School of Medicine and Nanomedicine, Yamaguchi, Japan

**Keywords:** hyperthermia, plasmacytoma, side population, bortezomib, Pim-2

## Abstract

Multiple myeloma (MM) remains incurable, and MM-initiating cells or MM progenitors are considered to contribute to disease relapse through their drug-resistant nature. In order to improve the therapeutic efficacy for MM, we recently developed novel superparamagnetic nanoparticles which selectively accumulate in MM tumors and extirpate them by heat generated with magnetic resonance. We here aimed to clarify the therapeutic effects on MM cells and their progenitors by hyperthermia. Heat treatment at 43°C time-dependently induced MM cell death. The treatment upregulated endoplasmic reticulum (ER) stress mediators, ATF4 and CHOP, while reducing the protein levels of Pim-2, IRF4, c-Myc and Mcl-1. Combination with the proteasome inhibitor bortezomib further enhanced ER stress to potentiate MM cell death. The Pim inhibitor SMI-16a also enhanced the reduction of the Pim-2-driven survival factors, IRF4 and c-Myc, in combination with the heat treatment. The heat treatment almost completely eradicated “side population” fractions in RPMI8226 and KMS-11 cells and suppressed their clonogenic capacity as determined by *in vitro* colony formation and tumorigenic capacity in SCID mice. These results collectively demonstrated that hyperthermia is able to impair clonogenic drug-resistant fractions of MM cells and enhance their susceptibility to chemotherapeutic drugs.

## INTRODUCTION

Multiple myeloma (MM) still remains incurable despite the implementation of novel anti-MM agents, indicating the strong need for continued investigation to develop innovative strategies. MM-initiating cells or MM progenitors are considered to contribute to disease relapse through their drug-resistant nature, and therefore become among the most important targets in the treatment for MM [1]. MM cells not only reside in the bone marrow but also expand outside bone. Immature MM clonotypic cells or MM progenitors are rather preferentially localized in extraosseous niches and may eventually grow as extramedullary plasmacytoma [1, 2]. Extramedullary expansion of plasmacytoma is generally accepted to represent a high-risk condition with more aggressive features and poorer prognosis.

Superparamagnetic iron oxide nanoparticles (SPIONs), contrast media for magnetic resonance imaging (MRI), are able to time-dependently produce heat under an alternating current magnetic field arbitrarily set to cover the contrast-enhanced tumor lesions by SPIONs, which can be applied to magnetic hyperthermia treatment. Therefore, SPIONs potentially achieve what is called “theranostics”, a combination of tumor imaging and targeted treatment for tumors [3]. We developed novel SPION nanoclusters coated with folic acid and polyethylene glycol via the thiol-ene click reaction to improve their targeting efficiency toward tumors [4]. In mouse plasmacytoma models, the SPION nanoclusters preferentially and selectively accumulated in plasmacytoma tumors but not in the bone marrow via intravenous injection, and we were able to diagnose tumor localization by MRI with contrast enhancement and generate heat to selectively kill tumor cells [4, 5]. Subsequently, we have synthesized magnetic nanoparticle clusters covered by a thermoresponsive polymer containing anticancer drugs of interest, which can target tumors and release the drugs by melting the polymer membrane by heat [5]. We demonstrated that such nanoparticle clusters were able to effectively debulk tumors in plasmacytoma animal models though heating and locally releasing an anti-cancer drug in combination. In the present study, we therefore aimed to determine an effective combination of hyperthermia with anti-MM agents, and clarify the impact of hyperthermia on drug-resistant MM cells and their progenitors.

## RESULTS AND DISCUSSION

### Hyperthermia induces endoplasmic reticulum (ER) stress to cause MM cell death

**Figure 1:**
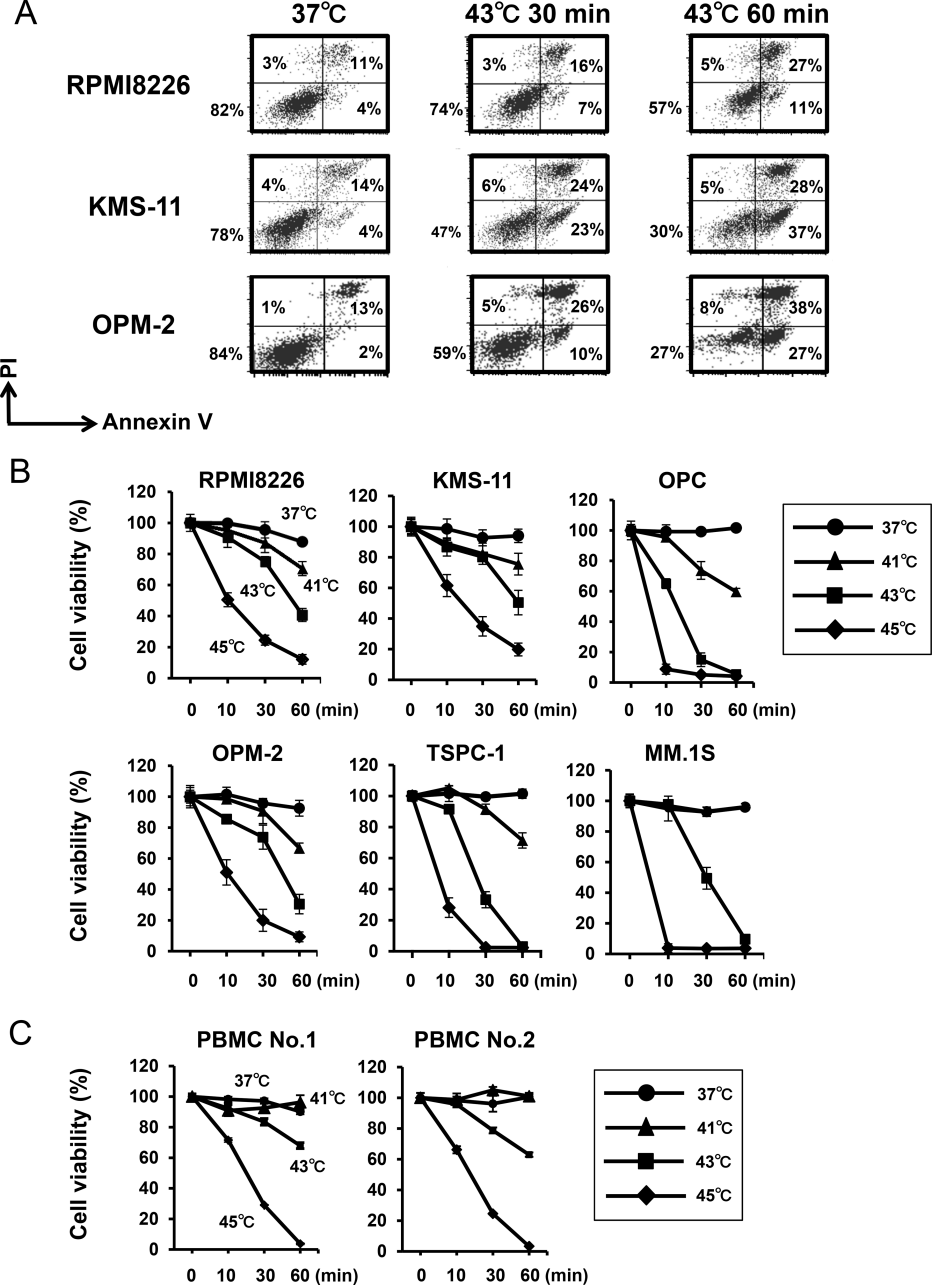
Hyperthermia induces MM cell death (**A**) RPMI8226, KMS-11 and OPM-2 cells were cultured at 37 or 43°C for the indicated time periods. After the heat treatment, the MM cells were further cultured at 37°C for 24 hours. The cells were stained with annexin V-FITC and propidium iodide (PI), and then analyzed by flow cytometry. (**B**) MM cell lines were cultured at 37, 41, 43 or 45°C for 10, 30 and 60 minutes. After the heat treatment, the MM cells were further cultured at 37°C for 24 hours. Cell viability was then analyzed by a WST-8 assay. (**C**) PBMCs from two normal donors were cultured at 37, 41, 43 or 45°C for 10, 30 and 60 minutes. Cell viability was then analyzed by a WST-8 assay.

The SPION nanoclusters we developed are able to heat within tumors without impairing non-tumorous tissues in plasmacytoma animal models [4, 5]. We therefore looked at the effects of heating at different temperatures up to 45°C. MM cells time-dependently underwent apoptosis when cultured at 43°C (Figure 1A). Hyperthermia induced MM cell death in a time and temperature-dependent manner (Figure 1B). Heat treatment at 45°C was able to induce substantial cell death in all MM cell lines even after 10 minutes. The heat treatment also reduce the viability of peripheral blood mononuclear cells (PBMCs) from normal donors, indicating impairment of normal cells by the heat treatment, although they seemed less sensitive to hyperthermia than MM cell lines at 41°C (Figure 1C). Therefore, because the heat treatment at 43°C appears to impair normal cells, heating should be localized within tumors to selectively kill tumors but spare the damage in surrounding normal tissues. In this regard, therapeutic options to deliver tumor-targeted heating such as “theranostics” with SPIONs appears to be beneficial.

**Figure 2:**
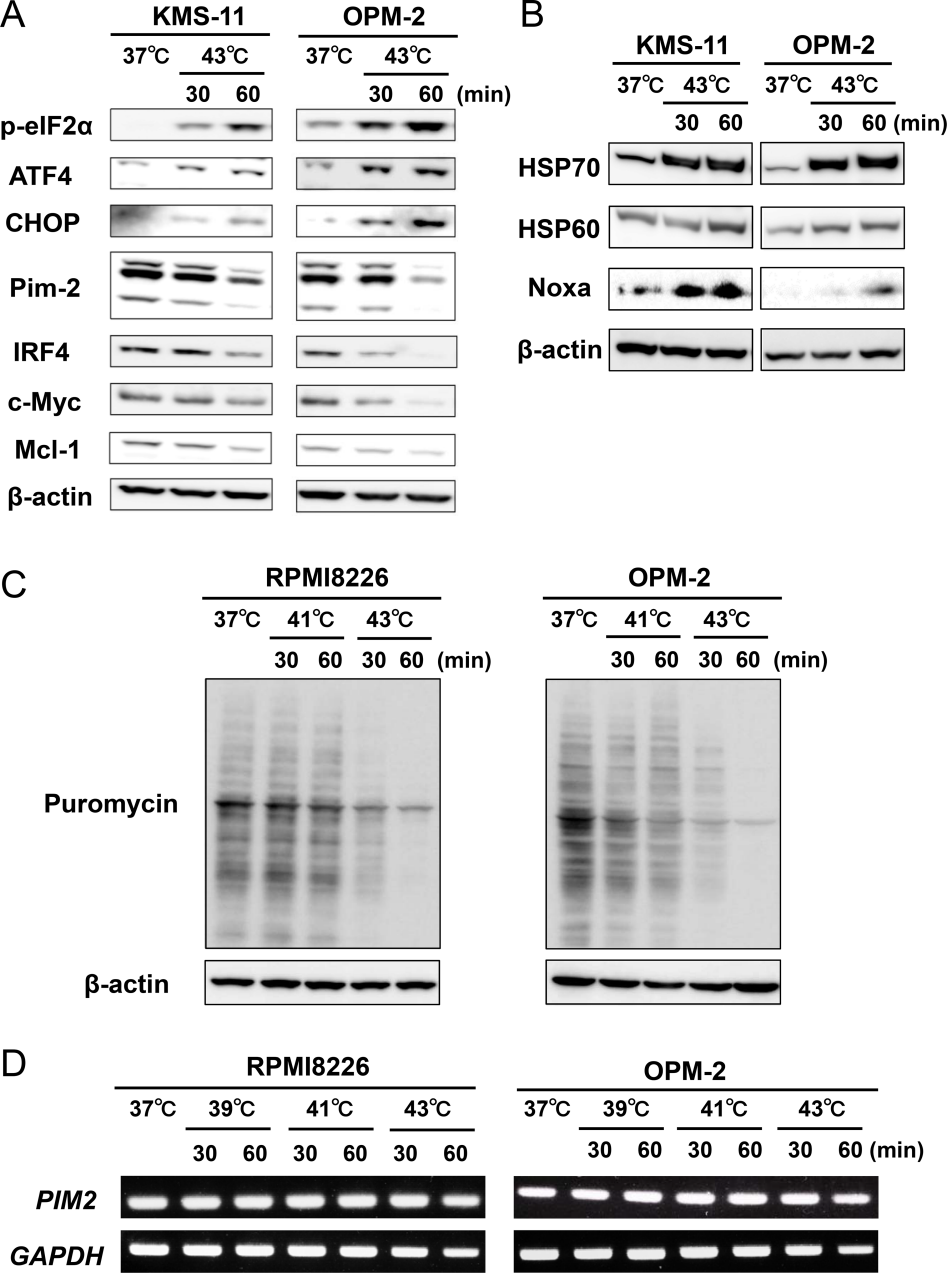
Hyperthermia induces ER stress along with the downregulation of IRF4, Pim-2, c-Myc and Mcl-1 in MM cells (**A**) KMS-11 and OPM-2 cells were cultured at 37 or 43°C for the indicated time periods. The cells were harvested, and the protein levels of phosphorylated eIF2α (p-eIF2α), ATF4, CHOP, IRF4, Pim-2, c-Myc and Mcl-1 were analyzed by Western blotting. β-actin was used as a protein loading control. (**B**) KMS-11 and OPM-2 cells were cultured at 37 or 43°C for the indicated time periods. The cells were harvested, and the protein levels of HSP70, HSP60 and Noxa were analyzed by Western blotting. β-actin was used as a protein loading control. (**C**) Puromycin incorporation. RPMI8226 and OPM-2 cells were treated with hyperthermia at 41 or 43°C for the indicated time periods. Puromycin was added at 1 μM for the last 15 minutes of the hyperthermia. The cells were harvested, and their puromycin incorporation was examined by Western blot analysis. β-actin was blotted as a loading control. (**D**) RPMI8226 and OPM-2 cells were treated with hyperthermia at 39 or 41 or 43°C for the indicated time periods. Then, the cells were incubated at 37°C for 6 hours. *PIM2* mRNA expression was determined by RT-PCR. *GAPDH* mRNA was used as an internal control.

After the heat treatment, eIF2α was phosphorylated along with up-regulation of its downstream ER stress mediators, ATF4 and CHOP, in MM cells (Figure 2A), indicating induction of ER stress. We further assessed the induction of ER stress in MM cells by hyperthermia. Heat treatment at 43°C up-regulated the protein levels of heat shock protein70 (HSP70) and heat shock protein60 (HSP60) along with Noxa in MM cells (Figure 2B). As shown in Figure 2A, the heat treatment at 43°C up-regulated in MM cells the phosphorylation of eIF2α, which leads to global suppression of translation. Therefore, we further investigated the effects of the heat treatment on translation in MM cells using puromycin incorporation. Puromycin incorporation in neosynthesized proteins directly reflects the rate of mRNA translation [6]. The heat treatment at 43°C time-dependently reduced the puromycin incorporation in MM cells, which is consistent with the phosphorylation of eIF2α and thereby global suppression of translation by the heat treatment (Figure 2C). Intriguingly, although tunicamycin, an inducer of ER stress, induced spliced *XBP1* mRNA, the heat treatment did not induce it (Supplementary Figure 1A). Such acute intense heat treatment may perturb the enzymatic activity responsible for *XBP1* splicing. We need to further look into the precise mechanism of the induction of ER stress in MM cells by hyperthermia.

Pim-2 is overexpressed in MM cells and regarded as a novel anti-apoptotic mediator for MM cells to be targeted [7, 8]. In parallel with the ER stress induction, the heat treatment reduced Pim-2 protein levels and Pim-2-driven survival factors, IRF4, c-Myc and Mcl-1 (Figure 2A). To dissect the mechanisms of the Pim-2 reduction, we first investigated the effects of the heat treatment on *PIM2* mRNA expression in MM cells. Heat treatment at 39, 41 and 43°C for up to 60 minutes only marginally affect *PIM2* mRNA expression (Figure 2D), although Pim-2 protein levels were apparently reduced by the heat treatment at 43°C for 60 minutes (Figure 2A). Pim-2 protein is reported to be quickly reduced in cells by ubiquitination-independent proteasomal degradation; thus Pim-2 protein levels in cells are mainly regulated by the rate of Pim-2 protein synthesis [9]. The heat treatment at 43°C markedly suppressed translation as indicated by the puromycin incorporation (Figure 2C). Therefore, potent suppression of translation by the heat treatment may at least in part cause the reduction of rapid-degrading Pim-2 protein in MM cells with marginally affecting its transcription levels.

### Bortezomib or the Pim inhibitor SMI-16a enhances MM cell death in combination with hyperthermia

**Figure 3:**
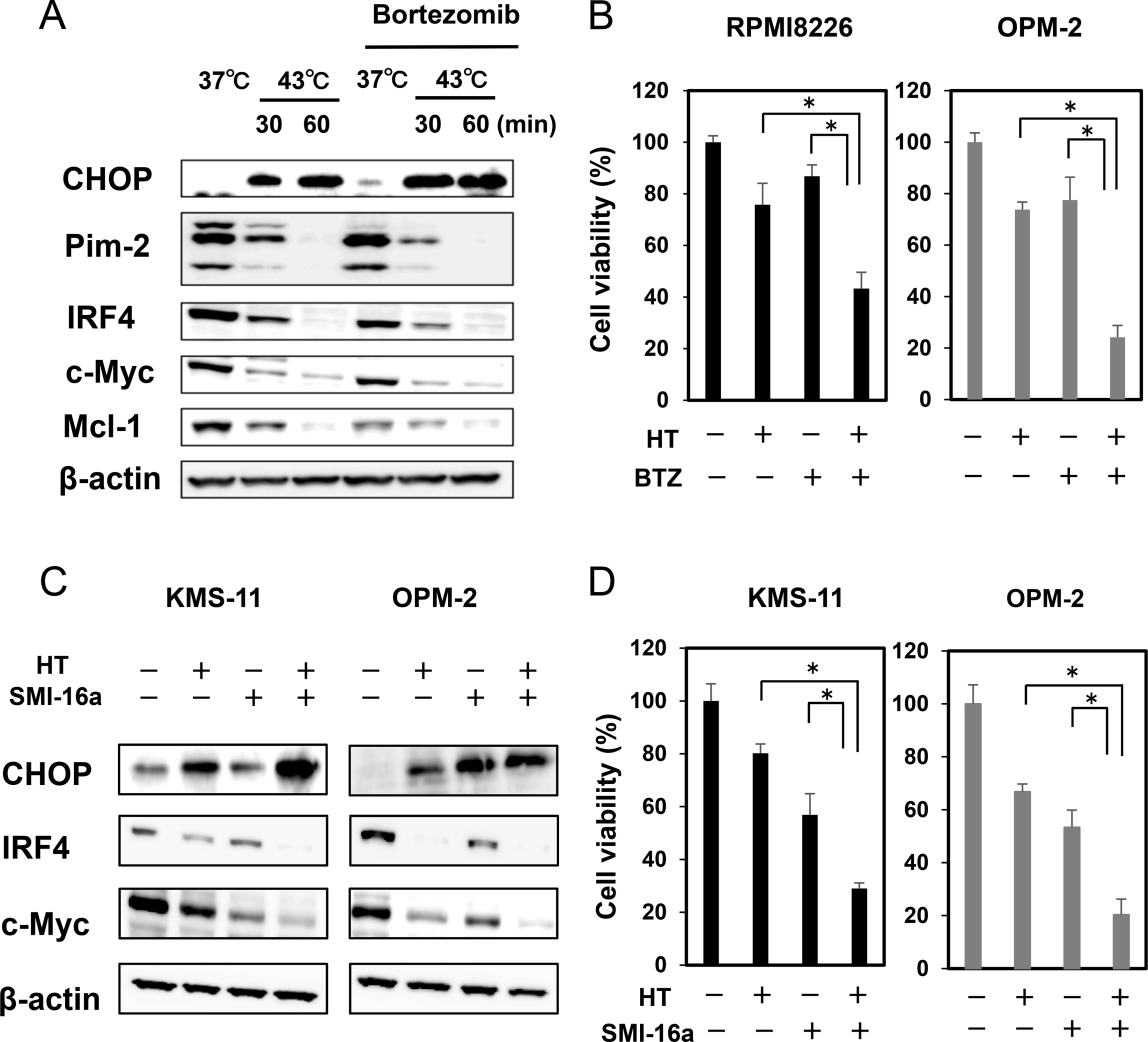
Hyperthermia and ER stress inducer, bortezomib or Pim inhibitor, cooperatively enhance MM cell death (**A**) OPM-2 cells were cultured at 37 or 43°C for the indicated time periods. The cells were then cultured at 37°C for another 24 hours. Bortezomib (BTZ) was added at 10 nM as indicated. The protein levels of CHOP, IRF4, Pim-2, c-Myc and Mcl-1 were analyzed by Western blotting. (**B**) RPMI8226 and OPM-2 cells were cultured at 37 or 43°C for 30 minutes. The cells were further cultured at 37°C for 48 hours. BTZ was added at 10 nM as indicated. Cell viability was analyzed by a WST-8 assay. ^*^p < 0.05. (**C**) KMS-11 and OPM-2 cells were cultured at 37 or 43°C for 30 minutes. The cells were further cultured at 37°C for 24 hours. The Pim inhibitor SMI-16a was added at 50 μM as indicated. The protein levels of CHOP, IRF4 and c-Myc were analyzed by Western blotting. (**D**) KMS-11 and OPM-2 cells were cultured at 37 or 43°C for 30 minutes. The cells were further cultured at 37°C for 48 hours. The Pim inhibitor SMI-16a was added at 50 μM as indicated. Cell viability was analyzed by WST-8 assay. ^*^p < 0.05. HT, heat treatment.

The proteasome inhibitor bortezomib, an ER stress inducer, was able to enhance the induction of CHOP and further suppress the protein levels of IRF4, c-Myc and Mcl-1 in combination with heat treatment at 43°C for 30 minutes, although bortezomib alone showed only slight effects in these experimental conditions (Figure 3A). Consistently, the heat treatment and bortezomib in combination cooperatively enhanced MM cell death (Figure 3B). To elucidate the mechanism of combinatory anti-MM effects of hyperthermia and bortezomib, we next examined accumulation of ubiquitinated proteins in MM cells upon treatment with hyperthermia or bortezomib alone or both in combination. Treatment with bortezomib substantially induced accumulation of ubiquitinated proteins in MM cells (Supplementary Figure 1B). However, ubiquitinated protein levels were only marginally affected in OPM-2 MM cells after the heat treatment at 43°C for 30 minutes. Furthermore, the heat treatment for 60 minutes rather reduced ubiquitinated protein levels even under addition of bortezomib. We further investigated the accumulation of ubiquitinated proteins in different MM cell lines. Although ubiquitinated protein levels were increased in RPMI8226 cells at 6 and 24 hours after the heat treatment at 43°C for 30 minutes, the ubiquitinated protein levels were rather reduced at 24 hours after the heat treatment at 43°C for 60 minutes (Supplementary Figure 1C, left). The heat treatment at 43°C did not induce the accumulation of ubiquitinated proteins at 6 hours, and tended to reduce ubiquitinated protein levels at 24 hours in KMS-11 and OPM-2 cells (Supplementary Figure 1C, right). These results imply the impairment of ubiquitination process by acute intense heating. It is plausible that heat treatment may perturb a protein synthesizing and modification process to cause accumulation of misfolded proteins and thereby ER stress without accumulation of large amounts of ubiquitinated proteins in MM cells. Mechanisms of the induction of ER stress appear to be different between heat treatment and bortezomib. The underlying mechanisms of the enhanced anti-MM effects by the combinatory treatment of acute intense heat treatment and proteasome inhibition seem not so simple, and need precise experiments, including ER-associated degradation and E3 ligase activity after the heat treatment.

Heat treatment reduced the protein levels of Pim-2, which may make MM cells more susceptible to Pim inhibition. Indeed, the Pim inhibitor SMI-16a almost completely eliminated Pim-2-driven survival mediators, IRF4 and c-Myc, in MM cells, which were already reduced by the heat treatment (Figure 3C). The combinatory treatment of heating with the Pim inhibition cooperatively increased MM cell death (Figure 3D).

### Hyperthermia minimizes “side population” and suppresses colony formation in MM cells

**Figure 4:**
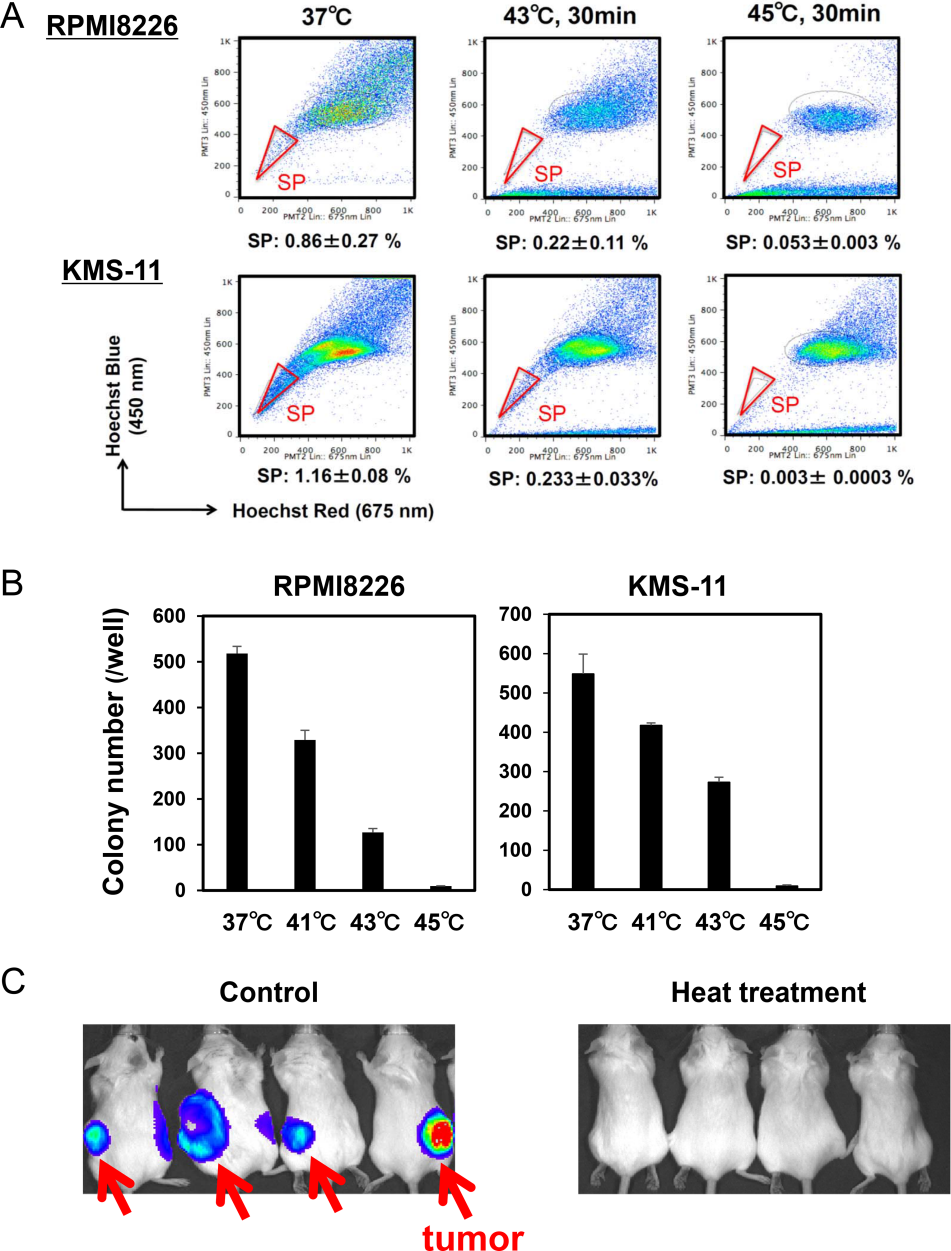
Hyperthermia impairs clonogenic MM cells (**A**) RPMI8226 and KMS-11 cells were cultured at 37, 43 or 45°C for 30 minutes. The cells were subsequently cultured at 37°C for 24 hours, and % distributions of SP fractions were determined by flow cytometry. (**B**) RPMI8226 and KMS-11 cells were cultured at 37, 41, 43 and 45°C for 30 minutes, and then subjected to methylcellulose assay. After culturing for 14 days, colony numbers were counted. (**C**) pGL4-transfected RPMI8226 were cultured at 37 or 43°C for 30 minutes, and then the MM cells were subcutaneously transplanted into SCID mice. The MM tumors were visualized at 6 weeks after the inoculation.

The SP is considered to be a drug resistant fraction containing cancer stem cells [10]. SP fractions were reported to be distinctly detected in the MM cell lines RPMI8226 and KMS-11 [11, 12]. Importantly, heat treatment at 43 and 45°C for 30 minutes markedly reduced the sizes of SP fractions in these MM cell lines although sizable non-SP fractions remained (Figure 4A), suggesting that SP cells can be targeted with heating. The heat treatment also suppressed colony formation of these MM cell lines in a temperature-dependent manner (Figure 4B). To further clarify the effects of hyperthermia on stemness or self-renewal capacity of MM cells, we next examined the effects of heat treatment on the tumorigenic capacity of MM cells using xenograft models. Luciferase-transfected RPMI8226 cells were treated with or without heat treatment at 43°C for 30 minutes, and then transplanted subcutaneously in SCID mice. The transplantation of MM cells without the heat treatment formed tumors in all the mice; however, no tumors formed in mice with inoculation of the heat-treated MM cells (Figure 4C). These results suggest that tumorigenic MM cells with a self-renewal capacity are susceptible to hyperthermia.

In summary, heat treatment is demonstrated to effectively enhance ER stress possibly through enhanced protein misfolding by heat, thereby inducing phosphorylation of eIF2α, along with the up-regulation of ATF4 and CHOP, to cause apoptosis in MM cells and their progenitors with stemness or self-renewing capacity. The enhanced ER stress may subsequently suppress translation of critical pro-survival mediators such as Pim-2, IRF4, c-Myc and Mcl-1, leading to further potentiation of MM cell death.

Hyperthermia was demonstrated in the present study to be able to effectively target SP fractions as well as MM progenitors with self-renewal capacity. MM cancer stem cells have been proposed to be responsible for drug resistance and relapse, although they have not been properly defined yet in MM [13]. Cancer stem cells are generally accepted to be dormant and resistant to a variety of chemotherapeutic drugs, and thereby cause an eventual relapse [14, 15]. Previous studies suggested that SP fractions may contain MM cancer stem cells [12, 16]. Given the existence of MM cancer stem cells in SP fractions in MM cells, hyperthermia may become a good therapeutic option to effectively target MM cancer stem cells.

We are currently developing new versions of mesoporous nanoparticles containing SPIONs, which can store anticancer drugs and selectively release them in tumor lesions after targeting tumors when heat is generated by magnetic resonance. In the present study, we demonstrated that the combination with the proteasome inhibitor bortezomib or the Pim inhibitor SMI-16a is able to potentiate heat-induced MM cell killing. Therefore, the above mesoporous nanoparticles carrying proteasome inhibitors or Pim inhibitors warrant further study, especially in the setting of drug-resistant extramedullary plasmacytomas. The strategy of “theranostics” with drug-containing and heat-generating nanoparticles can be also applied to malignant lymphoma such as NK cell lymphoma, and various solid cancers which remain resistant to conventional treatment modalities.

## MATERIALS AND METHODS

### Reagents

The following reagents were purchased from the indicated manufacturers: SMI-16a and mouse monoclonal antibody against Noxa from Calbiochem (Darmstadt, Germany); rabbit polyclonal anti-antibodies against human IRF4, bortezomib and mouse monoclonal antibody against C/EBP-homologous protein (CHOP), phosphorylated eukaryotic Initiation Factor 2α (p-eIF2α) and activating transcription factor 4 (ATF4) from from Cell Signaling Technology (Beverly, MA); Pim-2 and c-Myc from Abcam (Cambridge, UK); HSP70 from Biolegend (San Diego, CA); HSP60 from StressMarq Biosciences Inc.(Victoria, Canada); Mcl-1 from Medical and Biotechnological Laboratories (Nagoya, Japan); tunicamycin, puromycin and β-actin from Sigma (Saint Louis, MO); anti-puromycin antibody from Kerafast, Inc (Boston, MA); and anti-ubiquitin antibody was obtained from Millipore (Darmstadt, Germany).

### Cells and cultures

The human MM cell lines, RPMI8226, KMS-11 were obtained from the American Type Culture Collection (Rockville, MD, USA). TSPC-1 and OPC were established in our laboratory [17]. OPM-2 was purchased from the German Collection of Microorganisms and Cell Cultures (Braunschweig, Germany). MM.1S was kindly provided by Dr. Steven Rosen (Northwestern University, Chicago, IL, USA). PBMCs were isolated from normal subjects as described previously [17]. All procedures involving human specimens were performed with written informed consent according to the Declaration of Helsinki and using a protocol approved by the Institutional Review Board for human protection.

### Cell viability and apoptosis assays

Culture tubes with MM cell lines were tightly closed and immersed in a water bath at 41, 43 or 45°C for 10, 30 and 60 minutes. After the heat treatment, the MM cells were washed and incubated at 37°C 5%CO_2_ for 24 hours. Viable cell numbers were measured by cell proliferation assay using 2-(2-methoxy-4-nitrophenyl)-3-(4-nitrophenyl)-5-(2,4-disulfophenyl)-2H-tetrazolium (WST-8; Kishida Chemical, Osaka, Japan). To assess apoptotic cells, cells were stained with an annexinV-FITC and propidium iodide labeling kit (MEBCYTO Apoptosis Kit; MBL, Nagano, Japan) according to the manufacturer's instruction, and analyzed by flow cytometry.

### Side population (SP) analysis

SP fractions were analyzed as described previously [18, 19]. Briefly, cells were incubated at 1.0×10^6^ /ml with 5 μg/ml Hoechst 33342 (Sigma) for 90 minutes at 37°C in phosphate-buffered saline containing 3% fetal bovine serum, in the presence or absence of 100 mM verapamil (Sigma). After the incubation, the cells were washed and propidium iodide (1 mg/ml) was added to dead cells. SP fractions were analyzed with a flow cytometer (Beckman Coulter, Tokyo, Japan).

### Colony assay

After heat treatment, MM cells were plated out in triplicates at 1 × 10^4^ cells per dish into H4034 methylcellulose medium (Stem Cell Technologies), and cultured for 14 days. The numbers of colonies with > 40 cells were then counted under an inverted microscope.

### Western blot analysis

Cells were collected and lysed in lysis buffer (Cell Signaling, Beverly, MA) supplemented with 1 mM phenylmethylsulfonyl fluoride and protease inhibitor cocktail solution (Sigma). The cell lysates were subjected to SDS-PAGE on a 10% polyacrylamide gel, and then transferred to polyvinylidene difluoride membranes (Millpore, Billerica, MA). The membranes were blocked with 5% non-fat dry milk in TBS with 0.01% Tween 20 for 1 hour at room temperature and incubated for 16 hours at 4°C with the primary antibodies. After washing, a secondary horseradish peroxidase-conjugated antibody was added and the membranes were developed using the enhanced chemiluminescence plus Western blotting detection system (American Biosciences, Piscataway, NJ).

### RT-PCR

Total RNA was extracted from cells using TRIZOL reagent (Gibco BRL, Rockville, MD). For reverse transcription-polymerase chain reaction (RT-PCR), 2 μg of total RNA was reverse-transcribed with Superscript II (Gibco) in a 20-μL reaction solution. One tenth of the RT-PCR products were used for subsequent PCR analysis with 24–30 cycles of 95°C for 30 seconds, 58°C for 30 seconds and 72°C for 30 seconds. The following primers were used: The primers used for RT-PCR were as follows: human Pim-2 sense 5′-GGTAAGGGATTGAGGATC-3′ and anti-sense 5′-TGGGGTATTGGAAGGAAAG-3′; human XBP1 sense 5′-CCTTGTAGTTGAGAACCAGG-3′ and antisense 5′-GGGGCTTGGTATATATGTGG-3′; human GAPDH sense 5′-TGTCTTCACCACCATGGAGAAGG-3′ and anti-sense 5′-GTGGATGCAGGGATGATGTTCTG-3′.

### Establishment of luciferase-transfected RPMI8226 cells

Transfection of pGL4 luc (Promega) into RPMI8226 cells was performed using Lipofectamine LTX (Invitorogen) reagents according to the manufacturer's protocol. A total of 2.5 μg of pGL4-luc in 200 μl Opti-MEM was mixed with 6 μl Lipofectamine LTX reagent for 15 minutes at an ambient temperature. The DNA-LTX complex was then added to 6-well culture dishes containing 10^6^ cells. The medium was replaced at 16 hours after transfection, and the cells were subcultured in a medium containing G418 Geneticin at a final concentration of 500 μg/ml for 2 weeks. The medium was replenished every 3 days. The drug-resistant colonies were selected.

### Animal model

Severe combined immunodeficiency (SCID) mice (Charles River, Tokyo, Japan) were injected with 100 μl anti-asialo GM1 antiserum (Wako, Osaka, Japan) 1 day before tumor inoculation to eradicate residual natural killer cells. Luciferase-transfected RPMI8226 cells were treated with or without heat at 43°C for 30 minutes, and then the MM cells were transplanted subcutaneously in SCID mice. Six weeks after the inoculation, the tumorigenic capacity was evaluated by IVIS imaging system (SPI Summit Pharmaceuticals International, Tokyo, Japan). Animal experiments were conducted under the regulation and permission of the Animal Care and Use Committee of Tokushima University, Tokushima, Japan (toku-dobutsu 11083).

### Statistical analysis

Data were expressed as mean ± standard deviation. The differences in the means between groups were compared using a one-way analysis of variance (ANOVA) with Scheffe's post hoc tests. *P* ≤ 0.05 was considered as a significant difference. All statistics were performed using the Statistical Package for Social Sciences (SPSS 13.0 for Windows; Chicago, IL).

## SUPPLEMENTARY MATERIALS FIGURE


