# Supplementary material for: Effective impairment of myeloma cells and their progenitors by hyperthermia
Source: Oncotarget. 2017 Dec 7;9(12):10307–16. doi: 10.18632/oncotarget.23121 (PMC5828190; doi:10.18632/oncotarget.23121)
Supplement: Supplementary file 1 [file oncotarget-09-10307-s001.pdf]

Effective impairment of myeloma cells and their progenitors by hyperthermia

SUPPLEMENTARY MATERIALS

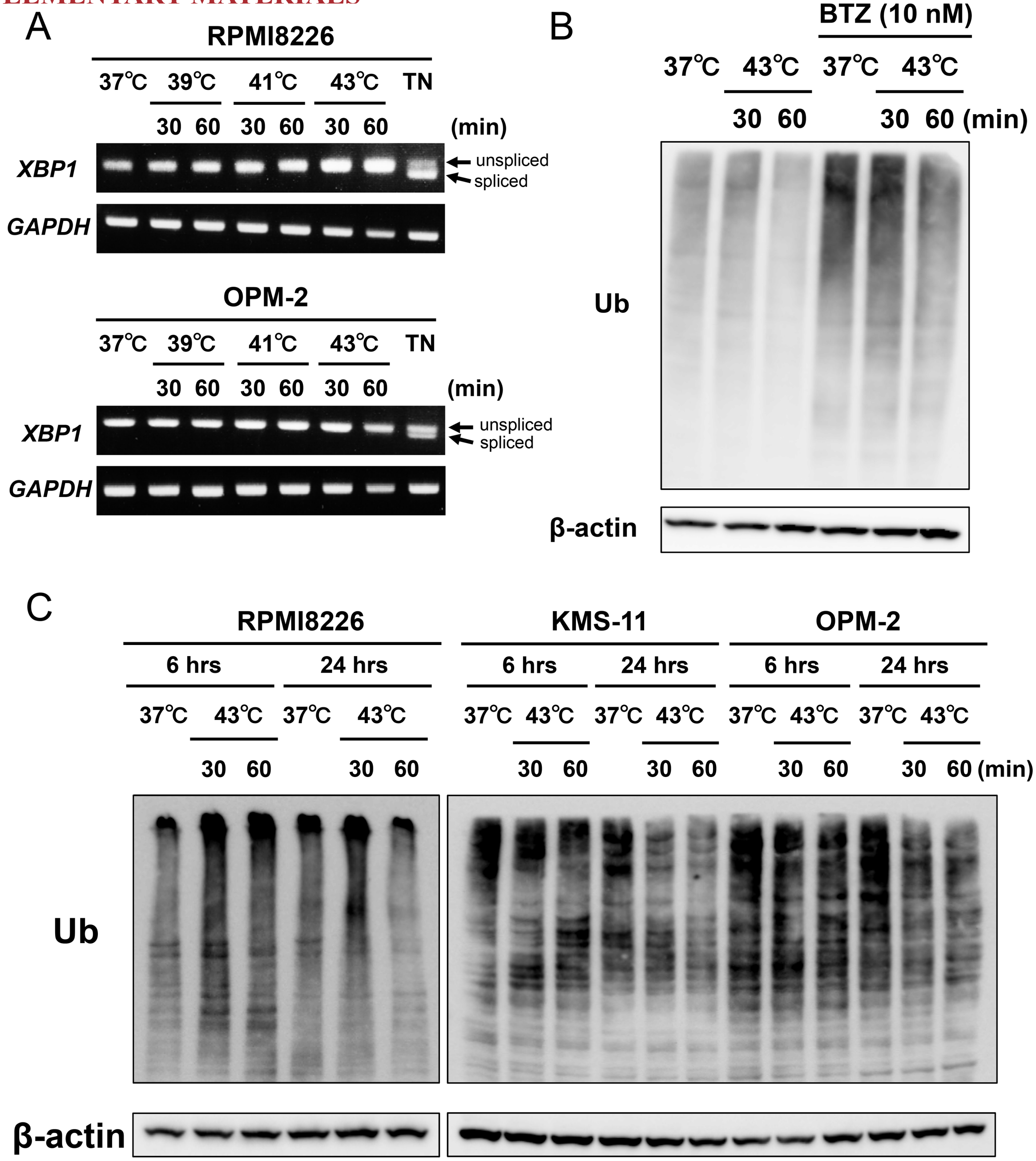

Supplementary Figure 1: (**A**) RPMI8226 and OPM-2 cells were treated with hyperthermia at 39 or 41 or 43°C for the indicated time periods. RPMI8226 and OPM-2 cells were also cultured with tunicamycin (TN; 5 μg/ml). Then, the cells were incubated at 37°C for 6 hours. Reverse transcription-PCR was done using a primer set that detects the unspliced (XBP1u, 442 bp) and spliced (XBP1s, 416 bp) form of *XBP1* mRNA. *GAPDH* mRNA was used as an internal control. (**B**) OPM-2 cells were cultured at 37 or 43°C for the indicated time periods. The cells were then cultured at 37°C for another 24 hours. Bortezomib (BTZ) was added at 10 nM as indicated. (**C**) RPMI8226, KMS-11 and OPM-2 cells were cultured at 37 or 43°C for the indicated time periods. After the heat treatment, the MM cells were further cultured at 37°C for 6 or 24 hours. The cells were harvested, and the accumulation of polyubiquitinated proteins levels (Ub) were analyzed by Western blotting. β-actin was used as a protein loading control.
